# Interventions for iron deficiency with or without anaemia in visceral surgery: recommendations for future research

**DOI:** 10.1016/j.bjao.2025.100503

**Published:** 2025-12-17

**Authors:** Caroline R. Evans, Henrik Kehlet, Sigismond Lasocki, Patrick Meybohm, Manuel Muñoz, Aryeh Shander, Donat R. Spahn, Suma Choorapoikayil, Kai Zacharowski

**Affiliations:** 1 Department of Anaesthesia and Intensive Care, University Hospital of Wales, Cardiff, UK; 2 Section of Surgical Pathophysiology, Rigshospitalet, Copenhagen University Hospital, Copenhagen, Denmark; 3 Département d’anesthésie réanimation, CHU Angers, Université d’Angers, Angers, France; 4 Department of Anaesthesiology, Intensive Care, Emergency and Pain Medicine, University Hospital Würzburg, Würzburg, Germany; 5 Perioperative Transfusion Medicine, Department of Surgical Sciences, Biochemistry and Immunology, School of Medicine, University of Málaga, Malaga, Spain; 6 Department of Anaesthesiology and Critical Care Medicine, Englewood Health, Englewood, NJ, USA; 7 University of Zürich, Zurich, Switzerland; 8 Goethe University, Department of Anaesthesiology, Intensive Care Medicine and Pain Therapy, University Hospital Frankfurt, Frankfurt, Germany

**Keywords:** haemoglobin, iron supplementation, safety, transfusion

Editor

Iron deficiency (ID) is the leading cause of anaemia. Numerous studies have shown the success of preoperative iron deficiency anaemia (IDA) management on improving patient outcomes.^1^, ^2^, ^3^ Preoperative iron supplementation protocols and outcome measures vary across studies, resulting in differences in inclusion criteria, study endpoints, and ultimately outcome data.^4^, ^5^, ^6^ Consequently, comparison between studies and the formulation of clear recommendations remain challenging. This is particularly relevant in visceral surgery, which involves both solid organ surgery and gastrointestinal surgery, placing patients at high risk for excessive intraoperative bleeding. In patients with gastrointestinal tumours, polyps, adenomas, and chronic inflammatory bowel disease, occult blood loss is frequent and may ultimately lead to ID.^7^, ^8^, ^9^ In this context, ID and anaemia are common owing to frequent blood loss. To enable meaningful comparison across studies and to generate conclusive evidence for clinical recommendations on preoperative ID treatment, consensus on patient selection, outcomes, and timing of intervention is needed. Therefore, we performed a narrative review of RCTs to evaluate the strengths and limitations of existing data on interventions for ID in the context of visceral surgery.

The literature search was performed between October and November 2023 using MEDLINE (PubMed), restricted to English language. The search included relevant Medical Subject Heading (MeSH) terms and free text terms (Supplementary material 1). We included RCTs comparing iron (i.v. or oral) with standard of care, placebo, or any active comparator (e.g. iron administered by a different route) in patients aged 18 yr or older, any sex, and undergoing visceral surgery of any kind. Patients were excluded from analysis if the intervention has been received ≥3 months after surgery.

We identified 517 records in PubMed published between 2014 and 2024, of which 12 studies^10^, ^11^, ^12^, ^13^, ^14^, ^15^, ^16^, ^17^, ^18^, ^19^, ^20^, ^21^ were RCTs comparing iron (i.v. or oral) with standard care, placebo, or any active comparator in patients (≥18 yr). Of the 12 studies, 1481 patients underwent various surgical procedures: liver surgery, abdominal surgery, curative tumour resection, colorectal cancer surgery, and post-bariatric abdominoplasty (Table 1 and Supplementary Table 1).

**Table 1:** Evaluation of the study design of included studies. ID, iron deficiency; IDA, iron deficiency anaemia; POD, postoperative day; TSAT, transferrin saturation. *Median with inter quartile ranges. ^§^Mean with standard deviation.

Publication	Study design	Type of surgery	*n*	Recruitment period	ID/IDA as an inclusion criterion	Time of randomisation and treatment (days)	Definition of ID
Assouline and colleagues^10^ (*Surgery* 2021; **170**: 813–21)	Single centre, double blind	Liver surgery	49	September 2015–August 2016	No	Recovery room	ID: ferritin <30 ng ml^−1^ and TSAT <20% Functional ID: ferritin >100 ng ml^−1^ and TSAT <20%
Froessler and colleagues^11^ (*Ann Surg* 2016; **264**: 41–6)	Single centre, open label	Major abdominal surgery	72	August 2011–November 2014	Yes	8 (6–13)∗	Ferritin <300 ng ml^−1^ or TSAT <25%
Fung and colleagues^12^ (*PLoS One* 2022; **17**: e0270640)	Single centre, double blind	Colorectal cancer surgery	40	August 2015–May 2020	Yes	23 (15–31)∗	Ferritin <30 ng ml^−1^ or 30–100 ng ml^−1^ with TSAT <20%
Keeler and colleagues^13^ (*Br J Surg* 2017; **104**: 214–21)	Multicentre, open label	Colorectal cancer surgery	116	May 2012–June 2014	No	21 (15–34)∗	Not specified
Khalafallah and colleagues^14^ (*Lancet Haematol* 2016; **3**: e415–25)	Multicentre, open label	Major abdominal surgery	201	September 2015–August 2016	Yes	POD 1	Ferritin ≤100 ng ml^−1^ or iron saturation ≤20%
Laso-Morales and colleagues^15^ (*Blood Transfus* 2022; **20**: 310–8)	Single centre, open label	Colorectal cancer surgery	104	September 2015–May 2018	No	POD 1	Ferritin <100 ng ml^−1^ and/or TSAT <20%
Montano-Pedroso and colleagues^16^ (*Lancet Haematol* 2018; **5**: e310–20)	Multicentre, open label	Post-bariatric abdominoplasty	56	April 2014–June 2016	Yes	POD 1	Ferritin <30 ng ml^−1^
Richards and colleagues^17^ (*Lancet* 2020; **396**: 1353–61)	Multicentre, double blind	Major abdominal surgery	487	January 2014–September 2018	No	15 (12–22)∗	Not specified
Talboom and colleagues^18^ (*Lancet Haematol* 2023; **10**: e250–60)	Multicentre, open label	Colorectal cancer surgery	220	October 2014–February 2021	Yes	5 (2–7)∗	TSAT <20%
Tayo and colleagues^19^ (*Nutrition* 2017; **33**: 113–7)	Single centre, open label	Myomectomy	66		No	3 weeks treatment before surgery	Not specified
Thin and colleagues^20^ (*Cureus* 2021; **13**: e17357)	Single centre, open label	Major abdominal surgery	30	November 2017–May 2018	Yes	11.5 (3.0)^§^	Ferritin <100 ng ml^−1^ or 100–300 ng ml^−1^ and TSAT <20%
Yagi and colleagues^21^ (*BMC Womens Health* 2022; **22**: 229)	Single centre, open label	Major abdominal surgery	40	October 2017–August 2021	No	39.7 (23.0)^§^	Not specified

We found substantial variation in population, interventions, and outcomes across the 12 studies. First, only a few studies used a double-blind design (*n*=3); most were open-label trials (*n*=9), likely attributable to the difficulty of creating a placebo that closely mimics i.v. iron treatment. In addition, many trials enrolled fewer than 100 patients over extended periods, limiting both external validity of the data and statistical power. Second, inclusion criteria varied widely (Supplementary Table 2): only five studies included patients with confirmed preoperative IDA, whereas seven enrolled either iron-deficient non-anaemic, anaemic, or non-anaemic patients. In these cases, unknown ID status makes it difficult to assess iron therapy effectiveness. Third, in some studies (*n*=4), i.v. iron was administered shortly before or after surgery which may be insufficient to elicit a haematological response. Importantly, erythrocyte maturation requires at least 4–6 days before changes become detectable in the blood. Therefore, to effectively raise preoperative haemoglobin (Hb) levels, i.v. iron should be given at least 7–14 days before surgery. Fourth, there is an ongoing debate about the appropriate cut-off values for diagnosing ID,^22^ which has persisted for decades. The WHO recommends a ferritin cut-off value of <15 ng ml^−1^ in healthy individuals and <70 ng ml^−1^ in individuals with infection or inflammation.^23^ The most commonly used definition to diagnose ID in surgical patients is currently ferritin <100 ng ml^−1^ or a transferrin saturation <20% in absence of inflammation (C-reactive protein [CRP] <5 mg L^−1^) and ferritin 100-300 ng ml^−1^ and transferrin saturation <20% in the context of inflammation (CRP >5 mg L^−1^).^24^ Given the low number of included studies in our analysis and the heterogeneity of the cut-off values used, as depicted in the Supplemental Table 2, it is not possible to recommend which cut-off values are the best to define ID. Finally, most studies used transfusion rates as a primary endpoint. However, transfusion rates and blood consumption are strongly influenced by institutional policies, clinical practices, and the extent of blood loss. Transfusion strategies were often undefined, and pre-transfusion Hb levels were rarely reported—representing a significant limitation. Additional factors such as bleeding control, coagulation monitoring, and coagulopathy management also affect transfusion requirements.^25^ Many studies did not report on the use of tranexamic acid or other relevant measures (e.g. hypothermia prevention), further limiting the interpretation of transfusion-related outcomes.

On the basis of these findings, we outline key considerations for future trials.

**Population:** Stangl and colleagues^26^ identified and rated outcomes for perioperative treatment of ID/IDA, highlighting the frequent lack of formal ID diagnosis before iron supplementation. Future studies should include patients with ID, with or without anaemia (Hb <130 g L^−1^ for both men and women), and stratify them to the severity of anaemia (mild, moderate, severe), treatment strategy, and relevant confounders. These confounders include inflammatory condition (CRP >5 mg L^−1^), red blood cell (RBC) transfusion, ongoing blood loss (e.g. chronic or drainage), diagnostic blood loss, coagulopathy, and supplementation with erythropoietin, folate, and vitamin B12. The target population should comprise patients undergoing major visceral surgery. Specific open procedures (e.g. gynaecological, oncological, pancreatic, liver, and possibly gastrectomy) should be listed and stratified accordingly.

**Intervention:** According to current guidelines for the management of preoperative anaemia, whenever possible, detection and classification of anaemia and ID should be accomplished at least 4 weeks before the scheduled surgical procedure.^27^ Treatment with oral iron is preferred in patients when there is sufficient time between diagnosis and surgery. However, patients with impaired gastrointestinal function, such as those with inflammatory bowel disease, are unlikely to benefit from oral iron owing to reduced absorption. In addition, patients with chronic blood loss may be unable to adequately replenish their iron stores with oral supplementation. In such cases, i.v. iron should be considered to achieve rapid iron repletion. We recommend i.v. iron supplementation in ID(A) patients at least 7–14 days before surgery to increase Hb levels before surgery.^28^^,^^29^ To enable adequate dosing, the Ganzoni formula should be used to determine the i.v. iron dose for supplementation.^30^ It is noteworthy that timely treatment allows re-assessment of iron stores and facilitates additional i.v. iron supplementation if required. Therefore, we recommend evaluating the presence of ID(A) 2–4 weeks before surgery. However, in cancer surgeries, where delays may affect survival chance, treatment timelines must remain feasible. For an RCT, only patients with confirmed ID should be included, and patients with combined deficiency such as folate or vitamin B12 should be excluded. This review did not aim to evaluate the effectiveness of erythropoietin, either as monotherapy or in combination with i.v. iron. In studies where erythropoietin is administered, this should be accounted for in the analysis to distinguish between single-agent and combination therapy, and to address patient heterogeneity.

**Outcomes:** We recommend the following primary endpoint (No. 1) and secondary endpoints (Nos. 2–6).(1) Because ID is associated with impaired erythropoiesis, the primary endpoint should focus on **replenishment of iron stores and an increase in Hb level,** which is defined as increase of the preoperative Hb level (>130 g L^−1^). Potential confounding factors, such as chronic blood loss, inflammation, other types of anaemia and associated therapy, co-medications, age, and timing of treatment, should be considered when analysing Hb response. (2) ID is often associated with impaired **quality of life**, including fatigue, headache, reduced concentration, and limited mobilisation. Therefore, patient-reported outcomes (PROMs) related to quality of life before and after treatment should also be analysed. (3) **Length of hospital stay** is a practical metric, but discharge decisions are often influenced by factors beyond the patient’s clinical readiness. A simplified endpoint, such as ‘days alive and out of hospital’, is gaining popularity, although it may likewise be affected by discharge decisions.^31^^,^^32^ (4) **RBC transfusion** is often used as a measure. However, to allow adequate comparison between studies, factors such as blood volume, transfusion threshold, and Hb levels before and after transfusion must also be documented. (5) **Readmission** because of surgery-related complications is a useful outcome but requires careful classification to minimise bias. (6) **Composite outcome** could include recovery indicators such as faster mobilisation, increased exercise capacity, and fewer complications.

**Proposed trial designs:** Future trials should aim to replenish iron stores and increase preoperative Hb levels, ideally to above 130 g L^−1^. Patients with ID (with or without anaemia) would be randomised to receive i.v. iron or placebo/control. Stratification by surgery type, preoperative treatment timing, and inflammatory condition would facilitate meaningful group comparisons. An implemented Enhanced Recovery After Surgery (ERAS) programme is a prerequisite.^33^

**In conclusion,** design improvements are crucial to overcoming the limitations of existing trials. Key refinements include stricter population criteria, optimised interventions, clearly defined outcomes, and more robust trial protocols. Implementing these recommendations will enable a more accurate evaluation of i.v. iron supplementation for ID/IDA in major (visceral) surgery, ultimately clarifying its impact and improving patient outcomes.

## Authors’ contributions

Substantial contribution to conception and design, acquisition of data: CRE, HK, SL, PM, MM, AS, DRS, KZ

Analysis and interpretation of data: all authors

Drafting the article or revising it critically for important intellectual content: all authors

Final approval of the version to be published: all authors

Agreed to be accountable for all aspects of the work thereby ensuring that questions related to the accuracy or integrity of any part of the work are appropriately investigated and resolved: all authors

## Funding

Cambridge—a division of Prime, Knutsford, UK; Foundation of Health, Patient Safety and Patient Blood Management.

## Declaration of interests

CRE has received honoraria for speaking roles and travel grants for PBM activities from Pharmacosmos and Pfizer. She is part of the guideline groups for anaemia management in the perioperative setting for CPOC and the British Society of Haematology. She is the principal investigator for ITACS and NOTACS RCTs in cardiac surgery.

HK has received speaker fee from Pharmacosmos.

SL has received grants, personal fees, and non-financial support from Pharmocosmos, Vifor Pharma, Masimo, and Pfizer outside the submitted work. SL is the chair of NATA.

PM’s department received research grants from the German Research Foundation (ME 3559/1-1, ME 3559/3-1, ME 6094/3-2), BMBF (01KG1815), and BMG (ZMVI1-2520DAT10E, ZMII2-2523FEP50A). PM received honoraria for scientific lectures from Biotest AG, CSL Behring GmbH, Haemonetics, Pharmacosmos GmbH, and Vifor Pharma GmbH. PM is member of the executive boards of the Health, patients safety, and PBM foundation and the Network for the Advancement of Patient Blood Management, Haemostasis and Thrombosis (NATA), and a member of the Working Group of the Scientific Advisory Board ‘Cross-sectional Guidelines for Therapy with Blood Components and Plasma Derivatives’.

MM has received honoraria for consultancies and scientific lectures from Pharmacosmos (Denmark), Pharmanutra (Italy), and Zambon (Spain).

AS has received consulting/speaking funds from Accumen, CSL-Vifor, Pharmacosmos, Octapharma, Pharmaniaga, I-Sep, Grifols, Baxter, and Lindis Corp.

DRS is chair of the ABC-Trauma Faculty, sponsored by unrestricted educational grants from Alexion Pharma Germany GmbH, Munich, Germany; CSL Behring GmbH, Marburg, Germany; and LFB Biomédicaments, Courtaboeuf Cedex, France. DRS is also the president of Alliance Rouge, Bern, Switzerland; CEO of Swiss-PBM-Consulting GmbH, Zurich, Switzerland; and a member of the Advisory Board of Saipient AG, Zurich, Switzerland.

DRS received honoraria/travel support for consulting or lecturing from Alliance Rouge, Bern, Switzerland; Danube University of Krems, Austria; European Society of Anesthesiology and Intensive Care, Brussels, Belgium; International Foundation for Patient Blood Management, Basel, Switzerland; Korean Society of Anesthesiologists, Seoul, Korea; Network for the Advancement of Patient Blood Management, Haemostasis and Thrombosis, Paris, France; Society for the Advancement of Blood Management, Mount Royal, NJ, USA; Alexion Pharmaceuticals Inc., Boston, MA, USA; AstraZeneca AG, Baar, Switzerland; Baxter AG, Glattpark, Switzerland; Bayer AG, Zürich, Switzerland; B. Braun Melsungen AG, Melsungen, Germany; CSL Behring GmbH, Hattersheim am Main, Germany, and Berne, Switzerland; CSL Vifor (Switzerland), Villars-sur-Glâne, Switzerland; CSL Vifor (International), St. Gallen, Switzerland; Celgene International II Sàrl, Couvet, Switzerland; Daiichi Sankyo AG, Thalwil, Switzerland; Haemonetics, Braintree, MA, USA; iSEP, Nantes, France; Novo Nordisk Health Care AG, Zurich, Switzerland; Octapharma AG, Lachen, Switzerland; Pharmacosmos A/S, Holbaek, Denmark; Pierre Fabre Pharma, Alschwil, Switzerland; Portola Schweiz GmbH, Aarau, Switzerland; Roche Diagnostics International Ltd, Reinach, Switzerland; Shire Switzerland GmbH, Zug, Switzerland; Werfen, Bedford, MA, USA; and Zuellig Pharma Holdings, Singapore, Singapore.

KZ, the Department of Anaesthesiology, Intensive Care Medicine & Pain Therapy of the University Hospital Frankfurt, Goethe University, received support from B. Braun Melsungen, CSL Behring, Fresenius Kabi, and Vifor Pharma for the implementation of Frankfurt‘s Patient Blood Management programme. KZ has received honoraria for participation in advisory board meetings for Haemonetics and Vifor and received speaker fees from CSL Behring, Masimo, Pharmacosmos, Boston Scientific, Salus, iSEP, Edwards, and GE Healthcare. He is the principal investigator of the EU-Horizon 2020 project ENVISION (Intelligent plug-and-play digital tool for real-time surveillance of COVID-19 patients and smart decision-making in Intensive Care Units) and Horizon Europe 2021 project COVend (Biomarker and AI-supported FX06 therapy to prevent progression from mild and moderate to severe stages of COVID-19). KS is a partner for EU Horizon 2023 project EDiHTA. KZ leads as CEO the Christoph Lohfert Foundation and the Health, Patient Safety & PBM Foundation.

SC received honoria for writing from Thieme.

The other authors declare that they have no conflicts of interest.
