# Supplementary material for: Interventions for iron deficiency with or without anaemia in visceral surgery: recommendations for future research
Source: BJA Open. 2025 Dec 17;17:100503. doi: 10.1016/j.bjao.2025.100503 (PMC12771078; doi:10.1016/j.bjao.2025.100503)
Supplement: Multimedia component 1 [file mmc1.docx]

# Supplementation Information 1: Search terms

The search included relevant Medical Subject Heading (MeSH) terms and free text terms: ("iron deficiencies"[Mesh] OR "anemia, iron-deficiency"[Mesh Terms] OR "iron deficiency anaemia"[All Fields] OR "iron deficiency anemia"[All Fields] OR ("anemia"[All Fields] AND "iron-deficiency"[All Fields]) OR ("anaemia"[All Fields] AND "iron-deficiency"[All Fields]) OR ("iron"[All Fields] AND "anemia"[All Fields]) OR ("iron"[All Fields] AND "anaemia"[All Fields])) AND (("abdomen/surgery"[Mesh] OR "patient blood management"[title/abstract] OR "surgery"[title/abstract] OR transplant*[title/abstract] OR "pre-operative"[title/abstract] OR "preoperative"[title/abstract]) AND ("colorectal cancer"[title/abstract] OR "gynecology oncology"[title/abstract] OR "gynaecology oncology"[title/abstract] OR "benign"[title/abstract] OR "appendectomy"[title/abstract] OR "cholecystectomy"[title/abstract] OR "colon"[title/abstract] OR "colon resection"[title/abstract] OR "bowel resection"[title/abstract] OR "hernia"[title/abstract] OR "kidney"[title/abstract] OR "renal"[title/abstract] OR "liver"[title/abstract] or "abdominal"[title/abstract] OR "gastric"[title/abstract] OR "spleen"[title/abstract]))

# Supplemental Table 1: Key details of included trials

| **Study** | **Type of treatment** | **Primary outcome** | **Reported results for primary outcome** | **Weakness/Limitations** |
| --- | --- | --- | --- | --- |
| **Assouline et al.** | FCM: ≤1,000 mg dose (15 mg kg^-1^) | Mean difference in plasma levels of Hb and Hct between treatment groups on postoperative day 7 | The mean hemoglobin level at POD 7 did not differ significantly between the intervention and control group (11.1 ± 1.8 g dL^-1^ and 10.4 ± 1.6 g dL^-1^) with a mean difference of +0.7 g dL^-1^ ([95% CI, -0.3 to +1.7], P =0.173). At POD 7, iron supplementation resulted in significantly higher levels of Ferritin and TSAT and lower level of sTfR and sTfR-f index in the intervention group. Functional iron deficiency was therefore only found in the control group. | The timing of the last blood sample collection on postoperative day 7 may have underestimated the effect of IV iron given 4 hours after surgery. |
| **Froessler et al.** | FCM: Preoperative dose of ≤1,000 mg (15 mg kg^-1^) and postoperative dose of 0.5 mg per 1 mL recorded blood loss, if blood loss was >100 mL, administered within 2 days of surgery | Incidence of allogeneic blood transfusions (treatment versus usual care) | A 60% reduction in blood transfusion was observed in the IV iron group compared with the usual care group (31.25% vs 12.5%). | The study was terminated early due to higher-than-expected rates of poor transfusion outcome in the usual care group. Lack of stratification by type of abdominal surgery. |
| **Fung et al.** | Iron isomaltoside: ≤1,000 mg dose (20 mg kg^-1^) before surgery and same dose 2 weeks after surgery if Hb was <100 g L^-1^ | Perioperative changes in Hb and ferritin between baseline (diagnosis date) to before surgery | The Hb and ferritin concentrations were both significantly higher in the iron isomaltoside group than the control group. Mean Hb change from baseline to before surgery was higher in the iron isomaltoside group (7.8, 95% CI: 3.2 to 12.3 g.l^-1^) than the control group (1.7, 95% CI: -1.9 to 5.3 g.l^-1^). The mean difference was 6.1, 95% CI: 0.3 to 11.8 g.l^-1^ (P = 0.040). The ferritin change from baseline to before surgery between groups was large in favour of the iron isomaltoside group (mean difference 296.9, 95% CI: 200.6 to 393.2 μg.l^-1^; P < 0.001). | In relation to the number of units of packed RBCs transfused during the perioperative period (secondary outcome), the volume transfused should be reported, as well as the Hb and blood volume of the patient. A proportion of patients had anaemia of chronic inflammation with ID; this may have affected the results. |
| **Keeler et al. Br J Surg 2017** | FCM: ≤1,000 mg dose for the first infusion, and a second infusion ≥7 days later to reach required total dose of 1,500 mg or 2,000 mg, depending on weight and Hb | Blood transfusions (volume and date of transfusion).  Three- and five-year overall survival (long-term follow-up) | There was no difference in volume of blood administered (P =0⋅841) or number of patients transfused (P =0⋅470). No significant difference in 5-year overall survival (HR 1.22, 95% CI 0.65–2.28, P = 0.522) or disease-free survival (HR 1.08, 95% CI  0.61–1.92, P = 0.79) was observed between oral iron and IV iron. | Patients were included based on a non-standard definition of anaemia; inclusion criteria did not cover iron parameters  The decision to transfuse was based on the clinical condition of the patient; the publication does not mention the use of a transfusion protocol.  The overall transfusion use was lower than anticipated, which may have made the study vulnerable to type II error.  During long-term follow-up, receipt of allogeneic blood transfusion since the initial trial may have affected long-term outcomes. |
| **Khalafallah et al.** | FCM: single dose of ≤1,000 mg (15 mg kg^-1^) postoperatively | Changes in Hb and ferritin at 4 weeks postoperatively, and the number of transfused units of blood required postoperatively until discharge (treatment versus usual care) | Baseline mean Hb was 105.5 g L^-1^ (SD 13.8) in the standard care group versus 106.2 g L^-1^ (11.9) in the FCM group, improving at 4 weeks to 121.5 g L^-1^ (SD 14.5) in the standard group and 130.1 g L^-1^ (11.3) in the FCM group (mean difference of 7.84 g L^-1^, 95% CI 3.79–11.9; p<0.0001). Fewer blood units were transfused in the FCM group (to one of  103 patients [<1%]) than in the standard care group (to five of 98 patients [5%]; incidence rate ratio 0.10; 95% CI  0.01–0.85; p=0.035). | Mixed surgery population: of 201 patients randomised, 156 patients (77.6%) had major orthopaedic surgery and 8 patients (3.98%) had ‘other’ operations, leaving only 37 patients of interest for the focus of this review (those who underwent abdominal surgery [n=19; 9.45%], gynaecological surgery [n=10; 4.98%], or urological surgery [n=8; 3.98%]) |
| **Laso-Morales et al.** | FCM: single 1,000 mg dose on postoperative Day 1 | Change in Hb from postoperative day 1 to postoperative day 30 (FCM vs IS) | There were no between-group differences in mean change in Hb from postoperative day 1 to postoperative day 30 (FCM: 2.5 g/dL, 95% CI: 2.1-2.9; IS: 2.4 g/dL, 95% CI: 2.0-2.8; p=0.52) | Patients were assigned to iron treatment if they presented with mild-to-moderate anaemia postoperatively (Hb <11 g dL^-1^) – ID does not appear to have been measured postoperatively and was not part of this inclusion criterion. In addition to this, mean haematological values measured on the day of surgery show that patients were not iron deficient at this point, according to the definition of ID provided. Hb was not monitored beyond 30 days; measurements beyond 30 days could have provided information on when anaemia was corrected. Inflammatory parameters (such as CRP), which could help guide postoperative anaemia treatment, were not studied. The publication concludes that a significantly higher infection rate was observed in patients treated with IS versus FCM (38% [n=19] versus 9.8% [n=5]). While the publication mentions that the study was not designed to compare infection rate, the patient numbers are too low to draw any solid conclusions (e.g., infection of the surgical wound was the case in 3 patients treated with IS versus 1 patient treated with FCM) |
| **Montano-Pedroso et al.** | IS: one 200 mg dose in the immediate postoperative period and one 200 mg dose on postoperative day 1.  Iron polymaltose complex: postoperative 330 mg oral dose (equivalent to 100 mg elemental iron) twice a day for 8 weeks | Difference in mean Hb on postoperative day 56 (IS vs oral iron) | Mean baseline Hb concentration was  in the oral group 12.71 g dL^-1^ (SD 1.06) and in the IV iron group 12.24 g L^-1^ (SD 1.09). By postoperative day 56 the Hb concentration was 12.54 g dL^-1^ (SD 1.18) in the oral group and 12.80 g dL^-1^ (SD 0.81) in the IV iron group. Mean diﬀerence was 0.26 g dL^-1^ (95% CI  –0.28 to 0.80; p=0.009 in favour of the intravenous group). The minimum clinically relevant difference in  concentrations was not reached. | No preoperative assessment of iron status. Adherence to oral iron was not monitored; observed between-group differences may be due to reduced adherence to oral iron |
| **Richards et al.** | FCM: single, preoperative dose of 1,000 mg 10–42 days before planned date of surgery | Risk of the composite endpoint of blood transfusion or death, and the number of blood transfusion episodes from randomisation until postoperative day 30 | Death or blood transfusion occurred in 67 (28%) of the 237 patients in the placebo group and 69 (29%) of the 237 patients in the IV iron group (RR 1.03, 95% CI 0.78–1.37; p=0.84). There were 111 blood transfusions in the placebo  group and 105 in the IV iron group (RR 0.98, 95% CI 0.68–1.43; p=0.93). There were no significant differences between the two groups for any of the prespecified safety endpoints. | ID was not part of the inclusion criteria (specific iron studies were only part of a predefined subgroup analysis). IV iron was used outside of its therapeutic indication, raising ethical concerns |
| **Talboom et al.** | FCM: ≤1,000 mg dose for the first infusion, and a second infusion one week later to reach required total dose of 1,500 mg or 2,000 mg, depending on weight and severity of anaemia | The proportion of patients whose Hb level normalised (≥7.5 mmol L^-1^ [12 g dL^-1^] for women and ≥8 mmol L^-1^ [13 g dL^-1^] for men) from the beginning of treatment to surgery | Normalisation of Hb at day of admission was reached in 14 (17%) of 84 patients treated intravenously and 15 (16%) of 97 patients treated orally (RR 1.08 [95% CI 0.55–2.10]; p=0.83), but the proportion of patients with normalised Hb significantly increased for the IV  treatment group at later timepoints (49 [60%] of 82 vs 18 [21%] of 88 at 30 days; RR 2.92 [95% CI 1.87–4.58];  p<0.0001). | Long recruitment time (Oct 2014–Feb 2021), during which clinical practice may have changed. Dosing schedule of oral iron (three 200 mg tablets daily from randomisation until the day before surgery). Some patients (n=20) received an incomplete or wrong dose of IV iron (mostly due to rescheduling of surgery to take place at an earlier date). The study may have been underpowered to detect statistical differences in clinical outcomes in the entire cohort. |
| **Tayo et al.** | Routine haematinics: 5 mg folic acid daily and 200 mg oral iron three times daily plus 500 mg sorghum bicolor extract daily | Increases in RBC indices over a 3-week period from baseline | Increases in RBC count in participants  in the test group were highly significant (P < 0.0002). | Inclusion criteria did not cover iron parameters; iron status of patients was not checked. The primary endpoint was powered for an evaluable sample size of 56 patients, but this target was not met (46 patients were analysed). |
| **Thin et al.** | FCM: single preoperative dose of ≤1,000 mg or 15 mg kg^-1^ | Time from recruitment to study intervention, recruitment rate, and the loss-to-follow-up rate | A total of 56 eligible patients were invited to participate in the trial. Twenty-six patients declined to participate mainly because they were not being able to choose the preferred study drug. The remaining 30 patients went  through randomization, and 15 patients were allocated into the intervention and 15 into the control arm. | Treatment at the latest time point (7 days before surgery) was too close to the date of surgery. Recruitment rate slowed towards the end of the study when the prescription of IV iron became part of routine care in the centre. The pilot study was underpowered to detect any significant differences in perioperative blood transfusion, length of stay, and complication rate. |
| **Yagi et al.** | Oral iron plus NYT: 100 mg dose of sodium ferrous citrate daily and 7.5 mg NYT granules daily, prescribed preoperatively at outpatient visits | Changes in Hb and on the CFS and VAS-A before and after treatment in the preoperative period | Hb significantly increased in both groups (iron supplementation group: 9.9 ± 0.8 g dL^-1^ vs. 11.9 ± 1.6 g dL^-1^; NYT group: 9.8 ± 1.0 g dL^-1^ vs. 12.0 ± 1.0 g dL^-1^). CFS (17.9 ± 10.2 vs.  8.1 ± 5.2) and VAS-A (56 mm (50–70) vs. 23 mm (6–48)) scores were significantly decreased only in the NYT group. | Small sample size (n=30 analysed); questionable whether the results are valuable from a statistical point of view, given the small sample size and lack of blinding. Patients with a variety of gynaecological diseases were included (heterogeneous population). The publication does not clearly define ID amongst the eligibility criteria. The publication notes that the level of fatigue and anxiety may differ between benign and malignant tumours, and potential observational bias could not be eliminated due to the small sample size. Availability of the product might be a limitation, as the use of NYT (a Japanese herbal medicine) is not applicable to all practices/countries. |

FCM=ferric carboxymaltose; Hb=haemoglobin; Hct=haematocrit; ID=iron deficiency; IDA=iron deficiency anaemia; IS=iron sucrose; IV=intravenous; NYT=Ninjin’yoeito; RBC=red blood cell; VAS-A=Visual Analogue Scale for Anxiety; FCM=ferric carboxymaltose; FDI=ferric derisomaltose; IS=iron sucrose; IV=intravenous; POD 7=postoperative day 7; CI=confidence interval; TSAT=transferrin saturation; sTfr=soluble transferrin receptor; HZ=hazard ratio; RR=risk ratio; CFS=Cancer Fatigue Scale

# Supplemental Table 2: Overview of various iron definitions used within the study, either before or after iron treatment

|  | **Assouline et al.** | **Frössler et al.** | **Fung et al.** | **Khalafallah et al.** | **Laso-Morales et al.** | **Montano-Pedroso et al.** | **Talboom et al.** | **Thin et al.** |
| --- | --- | --- | --- | --- | --- | --- | --- | --- |
| **Ferritin <30 μg/l and TSAT <20%** |  |  |  |  |  |  |  |  |
| **Ferritin >100 μg/l and TSAT <20%** |  |  |  |  |  |  |  |  |
| **Ferritin <300 μg/l** |  |  |  |  |  |  |  |  |
| **TSAT <25%** |  |  |  |  |  |  |  |  |
| **Ferritin <30 μg/l** |  |  |  |  |  |  |  |  |
| **Ferritin 11-100 µg/l** |  |  |  |  |  |  |  |  |
| **Ferritin 30–100 μg/l and TSAT <20%** |  |  |  |  |  |  |  |  |
| **Ferritin <100 μg/l** |  |  |  |  |  |  |  |  |
| **TSAT <20%** |  |  |  |  |  |  |  |  |
| **Ferritin <100 μg/l and TSAT <20%** |  |  |  |  |  |  |  |  |
| **Ferritin 100–300 μg/l and TSAT <20%** |  |  |  |  |  |  |  |  |
